# The Association of Inorganic Arsenic Exposure with Hypertension and High Blood Pressure Among African Caribbean Adults in Tobago

**DOI:** 10.3390/ijerph23040477

**Published:** 2026-04-09

**Authors:** Nusrat Jahan, Ryan K. Cvejkus, Natalie F. Price, Victor Wheeler, Patrick J. Parsons, Elizabeth J. Mullin, Austin A. Roberts, Joseph M. Zmuda, Allison L. Kuipers, Alison P. Sanders, Iva Miljkovic, Aaron Barchowsky

**Affiliations:** 1 Department of Environmental and Occupational Health, University of Pittsburgh School of Public Health, Pittsburgh, PA 15261, USA; nuj2@pitt.edu (N.J.); nfp9@pitt.edu (N.F.P.); aps109@pitt.edu (A.P.S.); 2 Department of Epidemiology, University of Pittsburgh School of Public Health, Pittsburgh, PA 15261, USA; rkc14@pitt.edu (R.K.C.); zmudaj@edc.pitt.edu (J.M.Z.); 3 Scarborough General Hospital, Scarborough 901124, Trinidad and Tobago; victorwheeler74@gmail.com; 4 Division of Environmental Health Sciences, Wadsworth Center, New York State Department of Health, Albany, NY 12237, USA; patrick.parsons@health.ny.gov (P.J.P.); elizabeth.mullin@health.ny.gov (E.J.M.); austin.roberts@health.ny.gov (A.A.R.); 5 Department of Environmental Health Sciences, College of Integrated Health Sciences, University at Albany, Albany, NY 12237, USA; 6 Departments of Medicine and Epidemiology & Biostatistics, Michigan State University, Grand Rapids, MI 49503, USA; kuiper28@msu.edu

**Keywords:** arsenic, hypertension, environmental exposure, effect modification, blood pressure, sex differences, Afro-Caribbean, Tobago

## Abstract

**Highlights:**

**Public health relevance—How does this work relate to a public health issue?**

- The contribution of environmental exposures to the increased burden of hypertensive disease in the African Caribbean population is not well understood.
- The findings highlight the need for age- and sex-specific cardiovascular health risk assessment in populations exposed to environmental arsenic.

**Public health significance—Why is this work of significance to public health?**

- Urinary arsenic exposure was associated with increased odds of hypertension among women and increased DBP and MAP among men in the Tobago Health Study.
- Age- and sex-specific effects were observed, with ΣAs being associated with hypertension among women in mid-life and with higher MAP among men in mid-life, while these associations were absent in older women and men.

**Public health implications—What are the key implications or messages for practitioners, policy makers and/or researchers in public health?**

- Environmental exposures may contribute to age- and sex-specific cardiovascular differences among African Caribbean populations.
- We observed evidence of effect modification by sex, where higher arsenic was associated with higher MAP among men and a null relationship among women.

**Abstract:**

Arsenic is among the most important environmental toxicants contributing to the global prevalence of hypertension. Multiple studies have reported a greater burden of hypertension among people of African ancestry, yet the contribution of environmental factors to this burden is unclear. Therefore, we investigated the association of arsenic exposure with increased risk of hypertension and high blood pressure in 965 Afro-Caribbean adults in the Tobago Health Study. Linear and logistic regression analyses were conducted for the total cohort and stratified by sex and age separately. We also examined potential effect modification by sex. Each unit (μg/L) increase in ΣAs was associated with 2% higher odds of hypertension in the total cohort and 4% higher odds among women. Higher ΣAs was associated with higher diastolic blood pressure and mean arterial pressure (MAP) among men. The associations of higher ΣAs with hypertension among women and with higher MAP among men were significant only in mid-life but not in older age. The data suggest effect modification by sex for the relationship between ΣAs and MAP in men. The findings suggest that exposure to inorganic arsenic contributes to age- and sex-specific patterns for greater risks of hypertension and high blood pressure among Afro-Caribbean adults.

## 1. Introduction

According to the World Health Organization (WHO) and recent reviews, approximately 300 million people in at least 100 countries are continuously exposed to arsenic at levels above the WHO provisional guideline value of 10 μg/L via drinking water [1,2]. Cardiovascular diseases (CVDs) are considered the most severe non-cancerous health consequences resulting from chronic environmental exposure to arsenic. A recent scientific statement from the American Heart Association (AHA) identified contaminant metals/metalloids, such as arsenic, as major risk factors for CVD and hypertension [3]. Arsenic exposure from food and drinking water remains a major public health challenge given its ubiquitous presence globally and emerging evidence of its harmful effects at lower exposure levels than previously recognized [3]. Inorganic arsenic is a naturally occurring toxicant that can be found throughout the earth’s crust and is released into the environment by natural phenomena, such as volcanic activity, tectonic plate movement, and weathering of rock, as well as anthropogenic sources such as drilled water resources, mining processes, and agriculture. This toxic metalloid exists in nature in both inorganic and organic forms with different biologically relevant oxidation or valence states, including trivalent (AsIII) and pentavalent (AsV) forms. Arsenic can enter the body through ingestion, inhalation, or skin contact, but the most common source of exposure is drinking contaminated water. It can also come from certain foods, such as agricultural products and fish. Arsenic exposure can affect almost every organ system in the body and has been linked to a range of health problems, including skin lesions, cancer, diabetes, and lung disease [4].

Hypertension, often regarded as the most widespread risk for cardiovascular disease (CVD), is a significant contributor to premature mortality across the globe [5,6,7]. The latest estimate from the WHO indicates that approximately 1.28 billion adults aged 30 to 79 years have hypertension, with two-thirds of this population residing in low- and middle-income countries [8]. Hypertension is a multifaceted disorder characterized by various underlying vascular, metabolic, and neurological mechanisms. Although there is evidence suggesting an association between arsenic exposure and hypertension, the relationship is inconsistent across various studies and populations [9]. Previous studies conducted in arsenic-endemic areas in Bangladesh, Taiwan, and Chile reported a link between arsenic exposure and hypertension, while other studies did not identify consistent associations [9,10,11,12]. Nonuniformities in the dose–response associations were observed in studies conducted in both low- and high-exposure areas [9,10,11,12]. These inconsistencies have been explained by small participant numbers, the heterogeneity across studies, and the absence of prospective evidence [9]. Some studies also had important methodological limitations, such as lacking a standard definition of hypertension or appropriate arsenic exposure markers [13].

Sex differences in cardiovascular and metabolic disease susceptibility linked to low-level arsenic exposures have been previously reported [13,14,15]. A large cohort study of men and women in a region of Italy with low to moderate levels of arsenic exposure found increased cardiovascular disease risk in men and increased risk of diabetes in women [16]. A study of Mexican children found significant sex-based differences in arsenic methylation indices between male and female children [14]. Another study investigating the variability in human metabolism of arsenic in cohorts from Mexico, China, and Chile found discernible gender differences not just among high-exposure groups, but also in low-exposure groups across all three populations [13]. A study on a rural Bangladeshi population found that chronic arsenic exposure was linked to lower blood DNA methylation, which might be linked to arsenic-related blood pressure, with pronounced reductions in females indicating sex-dependent susceptibility [15].

A higher prevalence of hypertension among people of African ancestry compared to other racial and ethnic groups has been reported in multiple studies [17,18,19,20]. A recent study observed that Black immigrants from the Caribbean differed from U.S.-born African Americans and Black immigrants from Africa in rates of high blood pressure, diabetes, and obesity [21]. The Caribbean nation of Trinidad and Tobago has a cardiovascular disease (CVD) mortality rate of 289 per 100,000, which is among the highest in the Americas [22]. A recent AHA statement highlighted the significant burden of CVD among African Americans, indicating higher rates of heart failure, sudden cardiac events, and strokes, along with earlier onset and greater prevalence of hypertension, diabetes, and obesity compared to Whites [23]. A study measuring the prevalence of self-reported hypertension and heart disease in the Tobago population found significant prevalence of hypertension (30.2%) and heart disease (8.2%), with hypertension displaying gender specificity towards women [24]. A recent study among middle-aged and older Afro-Caribbean men in Tobago reported a very high prevalence of hypertension (62% of men had hypertension) and found that it was significantly associated with greater pulse wave velocity, a marker of arterial stiffness [25]. To our knowledge, no prior study has assessed the effects of arsenic exposure on blood pressure outcomes in an Afro-Caribbean population.

Given the unique nature of the cohort of individuals of direct African ancestry in the Tobago Health Study (THS) and the prevalence of hypertension in the cohort, we investigated whether exposure to arsenic contributes to their high prevalence of hypertension and elevated blood pressures in participants. Among these Tobagonian African Caribbeans, we anticipated that arsenic exposures originating from various sources would generally be low to moderate, as high endemic levels of arsenic have not been reported for the island. However, significant sources of arsenic in Tobago include commonly eaten shark species that bioaccumulate inorganic arsenic [26], a high consumption of poultry shown to contain arsenic [27], atmospheric dust from the Sahara Desert [28], extinct volcanic activity on the eastern portion of the island, and potential industrial and agricultural activities. Furthermore, intermittent exposure could arise from the use of hypopigmentation products, which have been found to contain significant amounts of arsenic [29]. We hypothesized that exposure to inorganic arsenic contributes to the greater burden of hypertension and elevated blood pressure changes afflicting the Afro-Caribbean population of Tobago. We tested our hypothesis in a cross-sectional analysis of Tobago Health Study participants by comparing urinary arsenical measures with well-documented measures of clinical health status, demographics, and lifestyle factors.

## 2. Materials and Methods

### 2.1. Participants

This study included a total of 965 Afro-Caribbean adults, comprising a subset of 499 men and 466 women from the Tobago Health Study [30]. The Tobago Health Study is a longitudinal, comprehensive health study initially launched with Afro-Caribbean men in 1998 [31], with women’s health being included starting in 2019. Participants for the current analysis were a random sample aged between 40 and 87, recruited from men attending an ancillary study of ectopic adiposity and health (2014–2017) and women attending their baseline visits (2019–2022). Eligibility criteria for both sexes included being ambulatory, noninstitutionalized, and free from terminal illnesses. Interviewer-administered health history questionnaires were collected. The study leveraged the collection of an extensive clinical dataset in the unique population of African ancestry individuals with low genetic admixture (6% non-African genetic ancestry) [32]. Approval for the study was granted by the Institutional Review Boards of both the University of Pittsburgh and the Tobago Ministry of Health and Social Services. All participants provided written informed consent prior to enrollment.

### 2.2. Study Questionnaire

Information on potential risk factors was obtained through structured questionnaires and physical examinations conducted by trained staff. Informed consent and questionnaires were collected on behalf of the participants in a face-to-face interview with trained clinic staff. Demographic variables included age and sex. Anthropometric measures included weight (kg), height (cm), average waist circumference (cm), and body mass index (BMI), calculated using height and weight (kg/m^2^). Height was recorded using a wall-mounted stadiometer to the nearest 0.1 cm, while weight was measured on a balance beam scale to the nearest 0.1 kg. Waist circumference was measured at the narrowest point of the waist, or at the level of the umbilicus if the narrowest point was not identifiable. Smoking status was categorized as never smoked, past smoker, and current smoker. Physical activity was assessed by self-reported weekly hours of walking. Family history of high blood pressure was assessed by asking participants whether any of their immediate blood relatives had ever been diagnosed with hypertension. Participants were asked to bring all prescription medications taken in the past 30 days, which interviewers recorded, and this information was used to identify the use of any antihypertensive medication.

### 2.3. Arsenical Speciation and Quantification

Morning spot urine samples were collected and stored at −80 °C until urinary arsenic species were measured at the Wadsworth Center’s HHEAR Trace Elements Laboratory. Arsenic species are reported to be stable at −80 °C for at least 30 years (Standard Reference Material, (SRM) 2669 Arsenic Species in Frozen Human Urine, National Institute of Standards and Technology (NIST), Gaithersburg, MD, USA). Note, however, the method of analysis used by the Wadsworth HHEAR laboratory deliberately forces the oxidation of arsenite (As^3+^) to arsenate (As^5+^) to negate any species interconversion in study samples between the time of collection and their storage at −80 °C. Urine samples (100 µL) were diluted to a 1.8 mL final volume using a reagent solution containing 4 mM tetrabutylammonium hydroxide, 2.5 mM succinic acid, 5% (*v*/*v*) high-performance liquid chromatography (HPLC) gradient-grade methanol in doubly deionized water and 30% (*w*/*w*) ultra-trace analysis-grade hydrogen peroxide (Sigma Aldrich, St. Louis, MO, USA) at pH 5.6. Diluted urine samples were vortexed and then centrifuged for 3 min at 14,100× *g* to separate particulates. An Agilent 1200 HPLC stack was used to separate five arsenic species on a ZORBAX SB 25 cm, 5 µm C-18 column. Arsenic species were then detected and quantified using an Agilent 8900 Inductively Coupled Plasma tandem Mass Spectrometer (ICP-MS/MS; Agilent, Santa Clara, CA USA). To maximize precision and accuracy, each batch of urine samples was analyzed along with bi-level internal quality control (IQC) materials and reagent blanks. Method accuracy was periodically verified against NIST SRM 2669, with found values within ±20% of the assigned values (Supplementary Table S1). Quality control procedures involved bracketing analytical-run analyses with two concentration levels of urine IQC materials that were previously characterized under conditions of repeatability. Within-run precision (%CV) was ≤5% for all species, except DMA (6.8%), while between-run precision varied from 3.4 to 7.7%. All elevated results for each element were repeated and confirmed, and 2% of urine samples were randomly selected for duplicate analysis. External QA/QC included analyses in four different External Quality Assessment Schemes (EQASs) and Proficiency Testing (PT) programs for arsenic speciation in urine. The five arsenic species quantified in urine included inorganic arsenic (iAs), monomethylarsonic acid (MMA), dimethylarsinic acid (DMA), arsenocholine (AsC), and arsenobetaine (AsB). Urinary arsenic species with a detection rate of >60% of samples were included in our analysis. Data are reported as the sum of urinary arsenic (ΣAs = iAs + MMA + DMA). The limit of detection (LOD) for arsenical species was 0.19 µg/L for AsC and AsB, 0.53 µg/L for DMA, 0.34 µg/L for MMA and 0.46 µg/L for iAs. The Limits of Quantitation (LOQ) and precision data for the As species are given in Supplementary Table S2. For statistical analysis, urinary arsenic levels below the LOD were assigned values of LOD/√2.

Urinary creatinine was measured by liquid chromatography tandem mass spectrometry (LC/MS/MS) using a well-validated method (Waters Acquity LC coupled to a Sciex API4000 MS/MS system, Milford, MA USA) to account for participants’ hydration status. Samples were prepared for analysis by diluting urine samples 1000-fold with deionized water and an isotopically labeled internal standard was added. Creatinine was separated from other matrix components on an Ascentis Express F5 column (100 × 2 mm, 2 µm) using an isocratic elution with 26:74 (5% methanol in water: acetonitrile—both supplemented with 0.1% formic acid). Quantitation was achieved using the stable-isotope dilution technique using creatinine standards in DI water. All but one value was above the method LOD (20 mg/dL), and its value was imputed as LOD/√2. These laboratory results were reviewed and approved according to the Wadsworth Center’s QA/QC policies to ensure that they conform to acceptable quality standards [33].

### 2.4. Clinical Measures

Blood pressure (BP) was measured using an automated oscillometer (Omron, Kyoto, Japan) with three readings recorded, with a 10 min seated rest in between each [34]. The average systolic (SBP) and diastolic blood pressure (DBP) were calculated from the second and third readings. Current antihypertensive medication use was recorded during participant interviews, as well as through detailed inventory of all medication, and models of BPs were adjusted to account for treatment effects. Although the 2017 ACC/AHA guidelines lowered the diagnostic threshold for hypertension to ≥130/80 mmHg [34], local healthcare providers in Tobago use the earlier guideline of a hypertension threshold of ≥140/90 mmHg to guide detection and treatment decisions. To ensure consistency with the clinical context in which participants were being evaluated and managed, we applied the 140/90 mmHg cutoff in this study. Participants receiving antihypertensive medication were classified as hypertensive regardless of measured blood pressure.

### 2.5. Statistical Analyses

All statistical analyses were conducted using R version 4.2.1. All measures were assessed for normality. Non-transformed urinary arsenic exposure measures were used in regression analysis since log transformation did not improve normality assessment. Urinary creatinine measures were log-transformed to stabilize variance and improve the adjustment for urine dilution in the models. Urinary arsenical (ΣAs) concentrations were categorized into exposure quartiles for both men and women. To assess the association between total arsenic (ΣAs) exposure quartiles and hypertension, we first created quartiles of ΣAs exposure by dividing the distribution of ΣAs into four equal groups of men and women separately. This ensured that quartile cutoffs reflected the arsenic exposure distribution within each sex, allowing for within-sex comparisons. Differences between the reference level (the lowest quartile) and higher quartiles were estimated with logistic (for HTN) regression models.

We investigated the cross-sectional associations of inorganic arsenic exposure with risk of hypertension and high blood pressure. We used multiple linear regression models to examine cross-sectional associations of urinary arsenicals with systolic blood pressure (SBP), diastolic blood pressure (DBP), pulse pressure (PP) and mean arterial pressure (MAP). Logistic regression models were used to examine odds of hypertension (HTN), defined as systolic BP ≥ 140, diastolic BP ≥ 90, or use of antihypertensive medication.

Our primary model was adjusted for log-transformed creatinine, age, sex (in total population models only), waist circumference, physical activity, and smoking status. We also provide results for another model, which includes additional adjustment for antihypertensive medication use along with all the covariates of the primary model described above. Linear regression models were used for continuous outcomes and logistic regression for the binary outcome (HTN).

Analyses were conducted among the whole sample population, as well as stratified by sex and age separately. For all models, beta coefficients (or odds ratios), 95% confidence intervals (CIs), and *p*-values (significance threshold used *p* < 0.05) were reported. Multiple comparison corrections were not applied because the analyses were based on predefined hypotheses. The same arsenic exposure variable (ΣAs) was evaluated consistently across all models and the outcomes examined are closely related blood pressure measures. Therefore, we report effect estimates with 95% confidence intervals and exact *p*-values to allow appropriate interpretation of the results.

We conducted sex-stratified analyses to estimate associations of arsenic exposure with blood pressure outcomes separately in men and women. The results are presented for the total population and the sex-stratified models separately. We further stratified the data by age in men and women by using the median age (58 years) of the total cohort as the cutoff, and the results are presented for mid-life (≤58 years) and older (>58 years) men and women separately. We also tested for effect measure modification (EMM) by sex across all outcomes using linear and logistic (for HTN) regression. The *p*-value for the interaction term is included with each model, which indicates whether the associations between arsenic exposure and each outcome differ significantly by sex.

## 3. Results

### 3.1. Baseline Characteristics

Baseline characteristics of the study population are summarized for the overall population and also stratified by sex to compare the differences among men and women in Table 1. The overall prevalence of hypertension was high (59.1%) and similar in men (57.3%) and women (61.5%). Men were, on average, older (60.3 vs. 55.7 years) and taller, while women had higher BMI (31.0 vs. 27.8 kg/m). Antihypertensive medication use was common among men (35.5%) and women (31.5%). While not all hypertensive participants were taking antihypertensive medication, all participants taking medication were categorized as hypertensive. Lifestyle factors differed markedly. Men reported more walking hours and were more likely to consume ≥4 alcoholic drinks per week (15% vs. 1.7%). Smoking was also predominant among men (26% smokers vs. 2.8% in women). Overall, the findings show behavioral and lifestyle characteristics within the study population, despite comparable hypertension prevalence.

### 3.2. Arsenical Exposure

**Figure 1:**
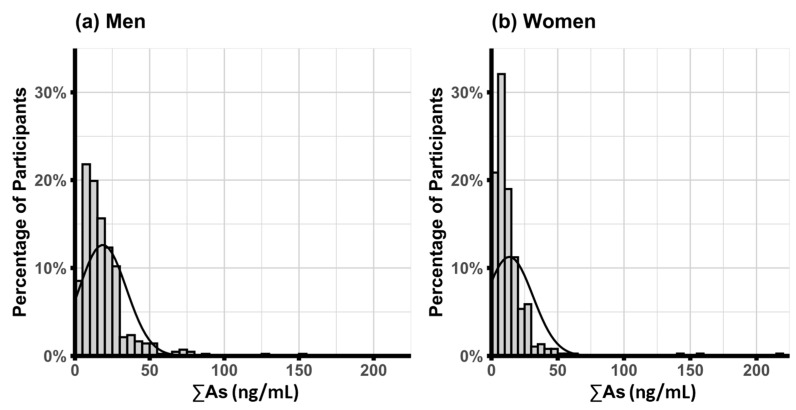
Frequency distribution of ΣAs among (**a**) men and (**b**) women. The frequency distributions of urinary ΣAs are shown for men (left) and women (right) separately. A normal distribution curve based on the group-specific mean and standard deviation is overlaid on each histogram.

The frequency distributions of ΣAs concentrations among men (a) and women (b) are shown in Figure 1. The mean (SD) ΣAs concentration was 16.2 (16.9) ng/mL overall, with 13.5 (17.7) ng/mL in women, and 18.6 (15.8) ng/mL in men. The majority of exposures were in the low-to-moderate range relative to global arsenic exposures associated with cardiovascular diseases [35,36].

### 3.3. Associations of Urinary ΣAs with HTN and Blood Pressure Outcomes

In our primary regression models, we assessed the association of arsenic exposure with HTN, and blood pressure measures (Table 2) adjusted for log-transformed urinary creatinine, age, sex (for the total population only), waist circumference, physical activity and smoking. Each unit (μg/L) increase in ΣAs was associated with 2% higher odds of HTN (OR: 1.02, 95% CI: 1.01–1.04, *p*: 0.008) in the total cohort and 4% higher odds (OR: 1.04, 95% CI: 1.01–1.08, *p*: 0.027) among women, while men had 1% higher odds (OR: 1.01, 95% CI: 1.00–1.03, *p*: 0.120) but the association was not statistically significant among men (Table 2). Sex-stratified analyses indicated that higher ΣAs was associated with elevated DBP (β = 0.08, 95% CI: 0.001–0.15, *p* = 0.048) and MAP (β = 0.08, 95% CI: 0.01–0.15, *p* = 0.029) among men, whereas women trended towards higher PP (β = 0.087, 95% CI: −0.003–0.177, *p* = 0.057) (Table 2).

Age-stratified analysis showed that higher ΣAs was associated with 5% higher odds of HTN among mid-life women (OR = 1.05, 95% CI: 1.01, 1.09, *p* = 0.017). Similarly, the association of higher ΣAs with higher MAP among men was significant in mid-life men (β = 0.10, 95% CI: 0.01–0.20, *p* = 0.037). None of these associations were significant among older women and men (Table 3).

We observed positive trends between each unit of ΣAs with systolic blood pressure, but findings did not reach statistical significance overall nor in sex-specific models. After adjustment for antihypertensive medication, the blood pressure-derived associations with ΣAs were generally attenuated (Table 4). However, the association between ΣAs and mean arterial pressure among men (β = 0.07, 95% CI: −0.001, 0.14, *p*: 0.051) was significant even after adjustment for antihypertensive medication use (Table 4).

Potential nonlinearity in the associations between ΣAs and blood pressure outcomes was assessed using piecewise (segmented) regression. There was no evidence of nonlinearity or threshold effects. Piecewise regression estimated breakpoints at very low ΣAs values (~2.4 μg/L), with slopes before and after the breakpoint not being significantly different for any of the outcome variables (Supplementary Figure S1). Thus, there was no suggestion of nonlinearity in the association of ΣAs with blood pressure outcomes.

### 3.4. Arsenic-Associated Sex Differences in Hypertension Prevalence

**Figure 2:**
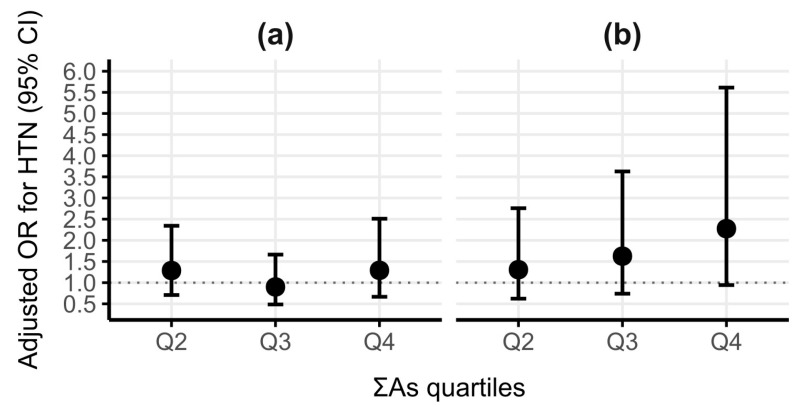
Adjusted odds ratios for hypertension across different exposure quartiles of urinary ΣAs in men (**a**) and women (**b**). Adjusted odds ratios for HTN from logistic regression analysis are shown comparing different exposure quartiles of urinary ΣAs—Q2, Q3, and Q4 to Q1 (reference; dashed line at OR = 1). All models were adjusted for log-transformed creatinine, age, waist circumference, physical activity, and smoking status. Left plot represents men; right plot represents women. Error bars indicate 95% CIs.

Figure 2 shows the adjusted odds ratios of HTN in association with different exposure quartiles of ΣAs in men and women separately. The odds rise steadily with increasing urinary arsenic exposure quartiles among women in the right, showing a clear dose–response pattern. However, among men, the odds ratios show no consistent trend across the exposure quartiles of ΣAs (Supplementary Table S1).

### 3.5. Effect Modification by Sex

We assessed effect measure modification (EMM) by sex and tested for interaction between ΣAs and sex. We observed suggestive evidence of effect modification by sex for the relationship between ΣAs and MAP (*p* interaction = 0.048) (Table 2), where the sex-stratified model demonstrated higher MAP among men and a null relationship among women, which remained significant even after adjustment for the antihypertensive medication use (*p* interaction = 0.015) (Table 3). We also observed similar suggestive evidence of effect measure modification by sex for the relationship between ΣAs and PP among women, where women had higher pulse pressure; however, the *p*-value for interaction was not statistically significant (*p*-interaction = 0.101) (Table 2).

## 4. Discussion

People of African ancestry are at increased risk of HTN and cardiovascular diseases [17,18,19,20], but the contribution of environmental exposures, such as arsenic, is often unrecognized. The Tobago Health Study cohort provides a unique opportunity to address this gap, as it includes a clinically well-characterized sample of middle-aged and older African-Caribbean men and women who are at increased risk of cardiovascular and metabolic diseases, relative to other Caribbean and global populations [21,22,23,24]. Specifically, we asked whether exposure to arsenic in Tobago could account for a portion of elevated blood pressure or greater odds of HTN.

In the Tobago cohort, greater ΣAs, a direct biomarker of exposure to inorganic arsenic, was associated with higher blood pressure parameters and greater odds of hypertension. The odds of HTN were particularly higher among women. Each unit (μg/L) increase in ΣAs was associated with 2% higher odds of HTN in the total cohort and 4% higher odds among women. These findings are consistent with our observation of low-to-moderate-arsenic-exposure effects on a Bangladeshi cohort [12]. However, the effects of low-to-moderate arsenic exposures on blood pressure and hypertension are not consistent across global populations [9,37,38], suggesting that the Tobago cohort is a relatively susceptible population.

Seafood is a significant source of iAs and inert arsenicals, such as arsenobetaine. Participants in the Tobago study consume a large amount of shark species that concentrate both iAs and arsenobetaine [26], necessitating the separation of arsenic species in the urine. Arsenobetaine levels averaged approximately 100–1000-fold higher than iAs, MMA, and DMA. However, arsenobetaine is inert and rapidly eliminated without toxicity. However, it is possible that the small amount of arsenobetaine that can be converted to DMA or the small amount of DMA that can be converted to arsenobetaine influenced the urinary level of DMA. Regardless, there were no significant associations of any individual arsenic species with blood pressures when evaluated individually, only the associations shown with ΣAs.

Sex-stratified models showed that ΣAs was associated with higher MAP and DBP only among men but not women. We observed suggestive evidence of effect modification by sex for the relationship between urinary ΣAs and MAP, with men having higher MAP, which remained significant even after adjustment for the antihypertensive medication use.

In the sex-stratified models including men only, we observed associations between arsenic and mean arterial pressure (MAP) and diastolic blood pressure (DBP), which may reflect increased peripheral vascular resistance [39,40]. The association between ΣAs and MAP among men remained significant even after adjusting for antihypertensive medication use. This persistent sex-specific pattern for MAP suggests that arsenic exposure may influence systemic vascular resistance more prominently in men than in women.

The median-age-stratified analysis showed that higher ΣAs was associated with 5% higher odds of HTN among middle-aged women (ages 40 to 58) but not participants over 58. Similarly, higher ΣAs was associated with higher MAP among middle-aged men only, but not participants over 58. Given the kinetics of arsenic, our measurement of urinary arsenicals reflects recent exposure to arsenic at the time of sampling. Since there were no age-dependent differences in the observed arsenic exposures, our observation of associations among individuals aged 58 and under but not older participants is intriguing. We posit that this may suggest a historic shift in exposures that occurred after the older population had passed a critical window of age-specific susceptibility to arsenic or that above a certain age, the effects of arsenic on hypertension and blood pressure may be negligible due to other unmeasured factors [41].

Our findings add to the growing body of evidence suggesting that arsenic may contribute to cardiovascular disease (CVD) risk, even at a low-to-moderate level of exposure [13,14,16,42]. This may reflect sex-based differences in hemodynamic regulation or vascular tone in response to arsenic exposure. Overall, these findings suggest that arsenic may affect cardiovascular function differently in men and women, potentially increasing pulsatile load more in women, while contributing to higher vascular resistance in men [39,40,43,44].

These observations are especially relevant given the high burden of CVD in Caribbean nations and among populations of African ancestry [21,22,23,24]. Our findings underscore the need to consider age, sex and environmental context when evaluating cardiovascular risk in populations of African ancestry. It is possible that hormonal, metabolic, or genetic factors influence how arsenic is metabolized and how it affects vascular function across sexes across the life course [41,45,46,47]. Additionally, sex differences in health behaviors, treatment patterns, or comorbid conditions could modify susceptibility [48,49,50,51].

The study limitations include the cross-sectional design of this study, which limits the ability to establish causality or temporal relationships between exposures and outcomes. There is a sampling time discrepancy between the male and female participants, which may potentially introduce bias, as lifestyle behaviors and environmental exposures may have changed over time. Lastly, behavioral factors (e.g., smoking and physical activity) were self-reported, which could introduce inaccuracies due to imperfect recall or a tendency to give socially acceptable responses. Additionally, since the THS cohort only included middle-aged and older adults between 40 and 87 years, the findings from this study may not be applicable to younger individuals of the Tobago population.

Despite these limitations, our study has several notable strengths. Our study is the first to explore the association of urinary arsenicals with HTN and high blood pressure among a middle-aged and older Afro-Caribbean population in Tobago. The THS has a very low loss to follow-up rate, and it continues to follow participants, and has also preserved biospecimens from prior visits, offering the opportunity to longitudinally assess environmental exposures in future studies. These preliminary findings lay the groundwork for longitudinal studies that can establish temporal relationships, thereby supporting stronger causal conclusions. The study included a fairly large number of participants (n = 965) with a relatively even split between men (n = 499) and women (n = 466), which allowed us to explore potential differences by sex. All arsenic measurements were carried out at the Wadsworth Center, New York State Department of Health, where strict quality control procedures and well-established laboratory methods were followed, giving confidence in the accuracy and reliability of the results (Supplementary Tables S1 and S2).

In sum, this study suggests that arsenic exposure is associated with higher blood pressure and hypertension in Afro-Caribbean adults. Observed patterns suggest that arsenic exposure may contribute to arterial stiffness and hypertension in women, and to systemic vascular resistance in men. These results highlight the need for further longitudinal studies to evaluate long-term cardiovascular impacts and to identify potential interventions that can reduce arsenic-related health disparities in high-risk populations.

## 5. Conclusions

In this well-characterized Afro-Caribbean cohort, we observed that arsenic exposure was linked to higher blood pressure and an increased risk of hypertension, and the patterns differed notably between men and women. Our findings indicate that inorganic arsenic exposure is associated with increased risk of hypertension in the THS participants. Overall, these results suggest age- and sex-specific susceptibility to arsenic-related hypertension. Our findings demonstrate sex-specific vascular responses to arsenic exposure. With greater arsenic exposure, women appear more susceptible to greater arterial stiffness and pulsatile pressure (PP) while men show greater systemic vascular resistance, reflected in elevated mean arterial pressure (MAP). These differences point to the possibility that biological sex influences how arsenic impacts cardiovascular function, potentially through variations in vascular tone, hormone regulation, or arsenic metabolism [9,45,52,53]. These findings suggest the need for age- and sex-specific approaches to assessing and preventing cardiovascular risks in people exposed to arsenic. Given the high burden of cardiovascular disease in Caribbean populations of African ancestry, these findings shed light on how environmental factors like arsenic exposure may contribute to hypertension risk. Even at low-to-moderate levels, arsenic might contribute to existing vulnerabilities, particularly in women. Our results highlight the importance of considering sex differences in environmental health research and point to the need for longitudinal studies to better understand the mechanisms behind these disparities. Public health strategies should address environmental exposures alongside traditional risk factors to more effectively reduce cardiovascular disease in Afro-Caribbean communities.

## Figures and Tables

**Table 1:** Characteristics of THS participants overall (n = 965) and stratified by sex.

Variable	Total Population n = 965	Men n = 499	Women n = 466
Hypertension status			
Normotensive	362 (40.9%)	213 (42.7%)	149 (38.5%)
Hypertensive	524 (59.1%)	286 (57.3%)	238 (61.5%)
Age (years)	58.1 ± 7.5	60.3 ± 5.0	55.7 ± 8.8
Height (cm)	170.0 ± 8.6	175.4 ± 6.4	164.2 ± 6.5
Weight (kg)	84.7 ± 15.8	85.6 ± 14.8	83.8 ± 16.9
BMI (kg/m^2^)	29.4 ± 5.3	27.8 ± 4.4	31.0 ± 5.7
Waist circumference (cm)	98.3 ± 12.1	98.0 ± 11.7	98.8 ± 12.5
Family history of hypertension	604 (63%)	274 (55%)	330 (71%)
Antihypertensive medication	311 (33.6%)	177 (35.5%)	134 (31.5%)
Walking hours in the past 7 days	2.2 ± 3.8	3.4 ± 4.7	1.0 ± 1.7
Nightly hours of sleep	6.5 ± 1.5	6.7 ± 1.5	6.4 ± 1.5
Drinking ≥ 4 drinks per week	83 (8.6%)	75 (15%)	8 (1.7%)
Smoking			
Never	820 (85%)	367 (74%)	453 (97%)
Past	96 (9.9%)	90 (18%)	6 (1.3%)
Current	49 (5.1%)	42 (8.4%)	7 (1.5%)

Continuous variables are presented as mean ± standard deviation (SD), and categorical variables as frequencies and percentages. Hypertension was defined as systolic BP ≥ 140 mmHg, diastolic BP ≥ 90 mmHg, or use of antihypertensive medication.

**Table 2:** Adjusted effect estimates and 95% confidence intervals (95% CIs) of associations between urinary arsenic with HTN (OR) and blood pressure outcomes (RR) for the overall THS population and stratified by sex.

Outcome	Predictor	Total Population		Men		Women		EMM by Sex
		Beta/OR (CI)	*p*-Value	Beta/OR (CI)	*p*-Value	Beta/OR (CI)	*p*-Value	Interaction *p*-Value
HTN	ΣAs	**1.02 (1.01, 1.04)**	0.008 **	1.01 (1.00, 1.03)	0.120	**1.04 (1.01, 1.08)**	0.027 *	0.488
SBP	ΣAs	0.09 (−0.01, 0.18)	0.065	0.07 (−0.07, 0.21)	0.335	0.1 (−0.02, 0.23)	0.107	0.618
DBP	ΣAs	0.04 (−0.01, 0.09)	0.090	**0.08 (0.001, 0.15)**	0.048 *	0.01 (−0.05, 0.08)	0.666	0.216
PP	ΣAs	0.04 (−0.02, 0.11)	0.214	−0.01 (−0.1, 0.09)	0.891	0.09 (0, 0.18)	**0.057**	0.087
MAP	ΣAs	0.02 (−0.02, 0.07)	0.349	**0.08 (0.01, 0.15)**	0.029 *	−0.02 (−0.08, 0.05)	0.607	0.048 *

Models were adjusted for urinary creatinine, age, sex (in total population models only), waist circumference, physical activity and smoking status. *p*-values for interaction were calculated using likelihood ratio tests for the effect measure modification (EMM) by sex. Significant differences are shown in bold. Hypertension (HTN), systolic blood pressure (SBP), diastolic blood pressure (DBP), pulse pressure (PP), mean arterial pressure (MAP). Sum of urinary As, ΣAs = (iAs + MMA + DMA). * *p* < 0.05, ** *p* < 0.01.

**Table 3:** Adjusted effect estimates and 95% confidence intervals (95% CIs) of associations between urinary arsenic with HTN (OR) and blood pressure outcomes (RR) for the overall THS population and stratified by age.

Outcome	Mid-Life Men (≤58)		Older Men (>58)		Mid-Life Women (≤58)		Older Women (>58)	
	β/OR (95% CI)	*p*-Value	β/OR (95% CI)	*p*-Value	β/OR (95% CI)	*p*-Value	β/OR (95% CI)	*p*-Value
HTN	1.01 (0.98–1.03)	0.733	1.02 (0.99–1.04)	0.129	**1.05 (1.01, 1.09)**	0.017 *	1.05 (0.99, 1.14)	0.229
SBP	−0.02 (−0.23, 0.19)	0.845	0.13 (−0.06, 0.33)	0.181	0.08 (−0.19, 0.35)	0.576	0.1 (−0.06, 0.27)	0.221
DBP	0.07 (−0.03, 0.18)	0.181	0.07 (−0.03, 0.18)	0.162	0.03 (−0.12, 0.19)	0.672	0.01 (−0.06, 0.08)	0.811
PP	−0.09 (−0.23, 0.05)	0.191	0.06 (−0.07, 0.19)	0.381	0.01 (−0.16, 0.19)	0.879	0.1 (−0.04, 0.23)	0.152
MAP	**0.1 (0.01, 0.2)**	0.037 *	0.06 (−0.04, 0.15)	0.27	−0.01 (−0.17, 0.14)	0.872	−0.02 (−0.1, 0.05)	0.534

Models were adjusted for urinary creatinine, age, sex (in total population models only), waist circumference, physical activity and smoking status. Mid-life (≤58 years) and older (>58 years). Hypertension (HTN), systolic blood pressure (SBP), diastolic blood pressure (DBP), pulse pressure (PP), mean arterial pressure (MAP). Sum of urinary As, ΣAs = (iAs + MMA + DMA). * *p* < 0.05 and significant differences are shown in bold.

**Table 4:** Adjusted values for antihypertensive medication.

Outcome	Predictor	Total Population		Men		Women		EMM by Sex
		Beta (95% CI)	*p*-Value	Beta (95% CI)	*p*-Value	Beta (95% CI)	*p*-Value	Interaction *p*-Value
SBP	ΣAs	0.06 (−0.03, 016)	0.189	0.05 (−0.09, 0.19)	0.471	0.08 (−0.05, 0.21)	0.237	0.749
DBP	ΣAs	0.025 (−0.02, 0.07)	0.306	0.064 (−0.01, 0.14)	0.085	−0.002 (−0.07, 0.06)	0.946	0.115
PP	ΣAs	0.037 (−0.03, 0.10)	0.263	−0.013 (−0.11, 0.08)	0.786	0.08 (–0.011, 0.17)	0.085	0.101
MAP	ΣAs	0.013 (−0.03, 0.06)	0.579	**0.07 (−0.001, 0.14)**	**0.051**	−0.03 (−0.09, 0.03)	0.371	0.015 *

Adjusted effect estimates and 95% confidence intervals (95% CIs) of associations between urinary arsenic and blood pressure outcomes (RR) for the overall THS population and stratified by sex. Models were adjusted for urinary creatinine, age, sex (in total population models only), waist circumference, physical activity, smoking status and antihypertensive medication use. *p*-values for interaction were calculated using likelihood ratio tests for the effect measure modification (EMM) by sex. Systolic blood pressure (SBP), diastolic blood pressure (DBP), pulse pressure (PP), mean arterial pressure (MAP). Sum of urinary As, ΣAs = (iAs + MMA + DMA). * *p* < 0.05 and significantly different values are designated in bold.

## Data Availability

Metal concentration data for this analysis are hosted and available through the Human Health Exposure Resource (HHEAR) Data Repository (https://hheardatacenter.mssm.edu), which has been approved under Icahn School of Medicine at Mount Sinai IRB Protocol #16-00947. Access to other study population data is available upon request, pending submission of an analysis plan and approval by the Tobago Health Study. Investigators interested in conducting data analyses should contact the Tobago Health Study PI, Iva Miljkovic (miljkovici@edc.pitt.edu).

## Supplementary Materials

The following supporting information can be downloaded at https://www.mdpi.com/article/10.3390/ijerph23040477/s1, Figure S1: Frequency distribution of ΣAs among mid-life (a) and older (b) are shown here separately. Overlaid on each histogram is a curve showing the expected normal distribution based on the group’s mean and standard deviation; Table S1: Method validation data based on analysis of NIST Standard Reference Material 2669 Toxic Elements in Frozen Human Urine; Table S2: Method Limits of Detection (LOD) and Limits of Quantitation (LOQ) for 5 arsenic species in urine calculated based on the IUPAC harmonized guidelines for a single laboratory validation; Table S3: Adjusted effect estimates and 95% confidence intervals (95% CI) of associations between urinary arsenic with HTN outcomes (OR) for the overall THS population and stratified by sex. Ref. [54] is cited in Supplementary Materials.

## Abbreviations

The following abbreviations are used in this manuscript:

HTN	Hypertension
SBP	Systolic Blood Pressure
DBP	Diastolic Blood Pressure
MAP	Mean Arterial Pressure
PP	Pulse Pressure
EMM	Effect Measure Modification
CVD	Cardiovascular Diseases
THS	Tobago Health Study
