# Supplementary material for: The Association of Inorganic Arsenic Exposure with Hypertension and High Blood Pressure Among African Caribbean Adults in Tobago
Source: Int J Environ Res Public Health. 2026 Apr 9;23(4):477. doi: 10.3390/ijerph23040477 (PMC13116142; doi:10.3390/ijerph23040477)
Supplement: Supplementary file 1 [file ijerph-23-00477-s001.zip › ijerph-4186741-supplementary.pdf]

Supplementary Material

Table S1. Method validation data based on analysis of NIST Standard Reference Material 2669 Toxic Elements in Frozen Human Urine.

	NIST SRM 2669 Level 1		NIST SRM 2669 Level 2	
	$X_{CRM} \pm U_{CRM}$	$X_{found} \pm U_{c,bias}$	$X_{CRM} \pm U_{CRM}$	$X_{found} \pm U_{c,bias}$
AsC (µg/L)	-	-	3.74 ± 0.35	3.39 ± 0.35
AsB (µg/L)	12.4 ± 1.9	13.4 ± 1.9	1.43 ± 0.08	1.51 ± 0.08
DMA (µg/L)	3.47 ± 0.41	3.16 ± 0.41	25.3 ± 0.7	25.1 ± 0.7
MMA (µg/L)	1.87 ± 0.39	1.72 ± 0.39	7.18 ± 0.56	6.66 ± 0.57
iAs (µg/L)	3.88 ± 0.32	3.91 ± 0.32	11.19 ± 1.0	11.4 ± 1.0

Notes: U_{CRM} is the expanded uncertainty given on the NIST CRM certificate of analysis (COA), with k = the coverage factor. For a confidence interval of approximately 95%, a coverage value of 2 was used (k = 2). $U_{c,bias}$ is the expanded uncertainty for the found value calculated using Equation 1, where n is the number of measurements, S_{val} is the standard deviation (SD) of the measurements, U_{CRM} is the expanded uncertainty given on the CRM certificate of analysis (COA), and k is the coverage factor[1]. For a confidence interval of approximately 95%, a coverage value of 2 is used (k = 2).

$$U_{c,bias} = k * \sqrt{\frac{S_{val}^2}{n} + (\frac{U_{CRM}}{2})^2} \qquad \text{Equation 1}$$

Table S2. Method Limits of Detection (LOD) and Limits of Quantitation (LOQ) for 5 arsenic species in urine calculated based on the IUPAC harmonized guidelines for a single laboratory validation.

	Method LOD (µg/L)	Method LOQ (µg/L)
Arsenocholine (AsC)	0.19	0.63
Arsenobetaine (AsB)	0.19	0.62
Dimethylarsinic acid (DMA)	0.53	1.76
Monomethylarsonic (MMA)	0.34	1.12
Inorganic arsenic (iAs) †	0.46	1.53

† Inorganic arsenic measured using hydrogen peroxide to oxidize any arsenite (As^{3+}) to arsenate (As^{5+}), thereby negating species interconversion.

Table S3. Adjusted effect estimates and 95% confidence intervals (95% CI) of associations between urinary arsenic with HTN outcomes (OR) for the overall THS population and stratified by sex.

Outcome	Predictor	Total Population		Men		Women	
		OR (CI)	P-value	OR (CI)	P-value	OR(CI)	P-value
	4th Quartile	1.1 (0.68, 1.78)	0.687	1.29 (0.71, 2.34)	0.409	1.28 (0.61, 2.71)	0.520
	3rd Quartile	1.1 (0.66, 1.82)	0.714	0.9 (0.49, 1.66)	0.734	1.65 (0.75, 3.72)	0.218
	2nd Quartile	1.57 (0.91, 2.71)	0.106	1.29 (0.67, 2.51)	0.449	2.43 (1, 6.08)	0.053

Models were adjusted for urinary creatinine, age, sex (in total population models only), waist circumference, physical activity, smoking status and anti-hypertensive medication use. Hypertension (HTN), systolic blood pressure (SBP), diastolic blood pressure (DBP), pulse pressure (PP), mean arterial pressure (MAP). Sum of urinary As, ΣAs = (iAs + MMA + DMA).

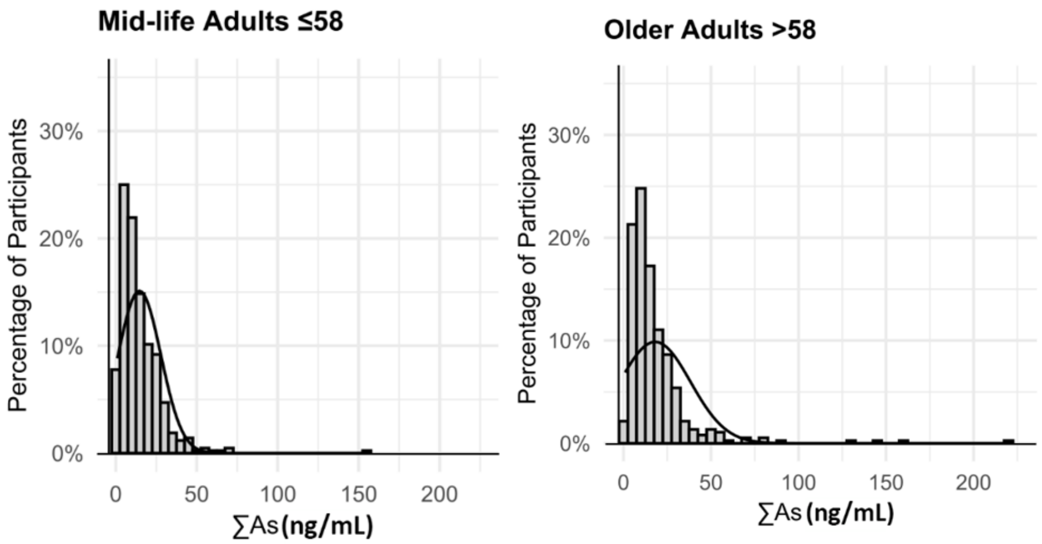

(**a**) (**b**)

Figure S1. Frequency distribution of ∑As among mid-life (**a**) and older (**b**) are shown here separately. Overlaid on each histogram is a curve showing the expected normal distribution based on the group's mean and standard deviation.

Reference

1. Sharpless, K.E.; Duewer, D.L. Standard Reference Materials for Analysis of Dietary Supplements. *Journal of AOAC INTERNATIONAL* **2019**, *91*, 1298–1302.
